# Epithelial Tissues Have Varying Degrees of Susceptibility to Kras^G12D^-Initiated Tumorigenesis in a Mouse Model

**DOI:** 10.1371/journal.pone.0016786

**Published:** 2011-02-02

**Authors:** Kevin C. Ray, Kayla M. Bell, Jingbo Yan, Guoqiang Gu, Christine H. Chung, M. Kay Washington, Anna L. Means

**Affiliations:** 1 Department of Surgery, Vanderbilt University Medical Center, Nashville, Tennessee, United States of America; 2 Department of Cell and Developmental Biology, Vanderbilt University Medical Center, Nashville, Tennessee, United States of America; 3 Department of Oncology, Johns Hopkins University School of Medicine, Baltimore, Maryland, United States of America; 4 Department of Pathology, Vanderbilt University Medical Center, Nashville, Tennessee, United States of America; Sanford-Burnham Medical Research Institute, United States of America

## Abstract

Activating mutations in the Kras gene are commonly found in some but not all epithelial cancers. In order to understand the susceptibility of different epithelial tissues to Kras-induced tumorigenesis, we introduced one of the most common Kras mutations, Kras^G12D^, broadly in epithelial tissues. We used a mouse model in which the G12D mutation is placed in the endogenous Kras locus controlled by inducible, Cre-mediated recombination in tissues expressing cytokeratin 19 including the oral cavity, GI tract, lungs, and ducts of the liver, kidney, and the pancreas. Introduction of the Kras^G12D^ mutation in adult mouse tissues led to neoplastic changes in some but not all of these tissues. Notably, many hyperplasias, metaplasias and adenomas were observed in the oral cavity, stomach, colon and lungs, suggesting that exposure to products of the outside environment promotes Kras^G12D^-initiated tumorigenesis. However, environmental exposure did not consistently correlate with tumor formation, such as in the small intestine, suggesting that there are also intrinsic differences in susceptibility to Kras activation. The pancreas developed small numbers of mucinous metaplasias with characteristics of early stage pancreatic intraepithelial neoplasms (PanINs), supporting the hypothesis that pancreatic ducts have the potential to give rise pancreatic cancer.

## Introduction

Cancer is thought to arise from a series of genetic and epigenetic changes that confer oncogenic properties on a cell and its descendents (reviewed in [1]). A number of oncogenes can contribute to cancer initiation or progression but none have been found to be sufficient for tumor formation. Rather, other genes must be mutated, silenced or overexpressed, within the cell or its environment, for a tumor to form. Kras is a proto-oncogene that normally relays signals from a variety of transmembrane receptors to intracellular effectors that regulate processes such as proliferation, survival, and migration. While its activity is tightly regulated, somatic mutations occur that render its activity constitutive and thereby oncogenic (reviewed in [2]). Activating mutations in Kras are found in histologically normal tissues as well as in tumors [3] indicating that while these mutations naturally accumulate over the lifetime of an individual, they only rarely lead to tumor formation.

Underscoring the ability of Kras mutations to contribute to tumor formation, they are common in many epithelial tumors including cancers of the pancreas, colon, and lung but are rare in cancers of the oral cavity and stomach [4]. The underlying mechanism for this tissue-specificity remains unexplored. It is possible that different cell types have different susceptibilities to the neoplastic effects of mutant Kras or that different tissues are more or less subject to Kras mutation. Kras appears to play different roles in different cancers, suggesting there are tissue-specific susceptibilities to Kras mutation. For example, in the pancreas, Kras mutation is an early step in tumor initiation [5]. However, in the colon, tumor initiation is thought to arise largely through aberrant activation of the Wnt signaling pathway with Kras mutation playing a later role in tumor progression [6].

One of the strongest correlations between cancer and Kras mutation occurs in pancreatic ductal adenocarcinoma (PDAC) which has Kras mutations in approx. 95% of cases [7]. Mouse models have shown that introducing mutant Kras throughout the pancreas beginning in embryogenesis results in many pancreatic intraepithelial neoplasms (PanINs) [8] that are thought to be the precursors of PDAC [9]. When combined with loss of tumor suppressor genes, these PanIN lesions progress to invasive and metastatic PDAC [10], [11], [12]. The genetic tools used to activate Kras during pancreatic development target mutant Kras to all cells of the pancreas, including both acinar and duct cells of the exocrine pancreas, making ontogeny of tumors impossible to determine. Because PDAC in humans has morphologic and molecular markers of duct-like cells, it was long held that neoplastic transformation of normal pancreatic ducts gives rise to PDAC [13]. However, we and others have recently shown that pancreatic acinar cells are capable of changing their cellular identity, or transdifferentiating, into duct-like cells in response to growth factors or damage [14], [15], [16], complicating the issue of the origin of PDAC. Understanding how PDAC arises is important for identifying appropriate biomarkers of cancer development as well as identifying targets that might be relevant in the treatment of human disease.

We have recently developed a genetic tool that allows us to investigate the susceptibility of different epithelial tissues to Kras^G12D^-initiated tumorigenesis in adult mice. Because epithelial tumors of some tissues are frequently associated with Kras mutation and those of other tissues are rarely associated, we tested the hypothesis that some tissues are refractory to the effects of Kras mutation, as opposed to being susceptible to its effects but rarely undergoing its mutation, by introducing the mutation into multiple tissues. We found that Kras^G12D^ could induce neoplastic changes in multiple, although not all, assayed tissues. Susceptible tissues included those which in humans rarely have Kras mutations as well as those that frequently do. There was some correlation of susceptibility with exposure to the outside environment, suggesting that environmental factors may play a major role in promotion of Kras^G12D^-initiated tumors. However, this correlation was not uniform indicating that there are also intrinsic differences in how cells respond to Kras activation. Additionally, we demonstrate that pancreatic ducts are capable of giving rise to mucinous metaplasias with characteristic of early PanIN lesions.

## Results

### CK19^CreERT^ produces recombination in multiple epithelial tissues

Kras^G12D^ mutation was targeted to multiple epithelial tissues via the CK19^CreERT^ [17] and LSL-Kras^G12D^ [18] genetically engineered insertional alleles. The LSL-Kras^G12D^ allele cannot be expressed until Cre recombinase removes a transcriptional stop cassette, positioning the mutated coding sequence in line with the endogenous Kras promoter. CK19^CreERT^ encodes the Cre recombinase fused to a region of the estrogen receptor that has been mutated to bind tamoxifen. This ERT domain prevents Cre from entering the nucleus and inducing recombination until it binds tamoxifen. For these studies, tamoxifen was injected into adult mice, abrogating embryonic effects. The CreERT coding region was inserted in exon 1 of the Cytokeratin 19 gene, a structural protein found in many epithelial tissues. We have shown previously that CK19^CreERT^ can activate a loxP-flanked reporter in cells throughout the gastrointestinal tract and in ducts of the pancreas, liver, and kidney [17]. Thus, CK19^CreERT^ can activate Kras^G12D^ in cells of all these tissues.

**Figure 1:**
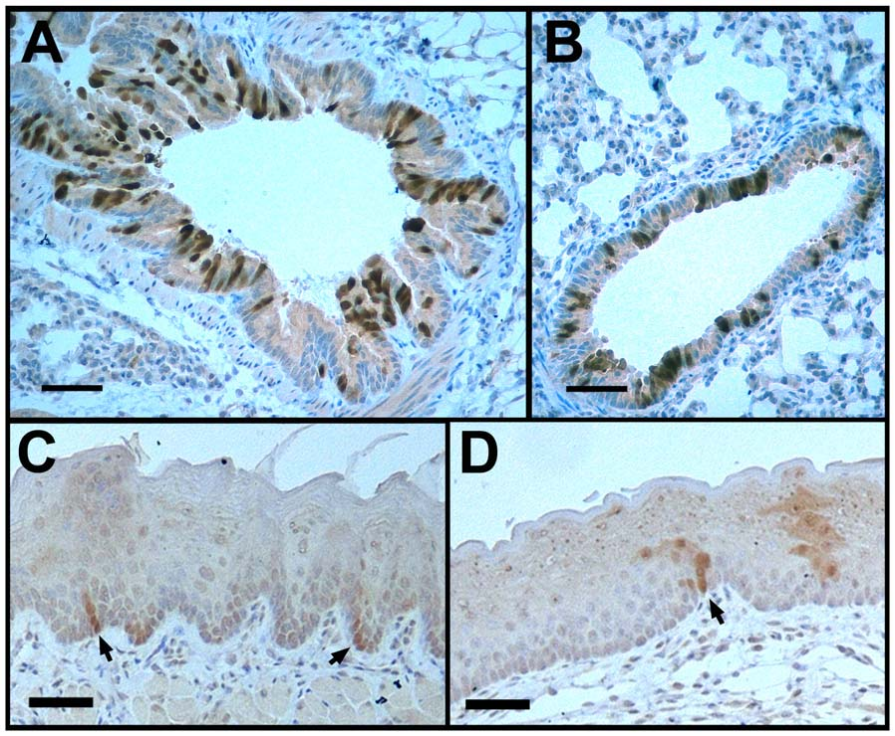
CK19^CreERT^ recombined an EYFP reporter in lung and oral cavity. Bronchus (A), bronchiole (B), lingual epithelium (C), and buccal epithelium (D) from CK19^CreERT^; R26R^EYFP^ mice immunolabeled for EYFP (brown). Arrows in C and D, labeled cells in basal layer of epithelia. Size bars, 50 µm.

In the course of these studies, we also found that CK19^CreERT^ was active in lung and oral cavity. In the lung, CK19 protein is widely expressed in all epithelium [19], [20]. However, we found that CK19^CreERT^ recombined the R26R^EYFP^ reporter largely in bronchi and bronchioles (28+/−10% of cells labeled) and rarely in alveoli (0.22+/−0.08% labeled) (n = 3 mice; Figure 1A, B and data not shown). CK19 protein is a minor component of oral epithelium [21]. Accordingly, when CK19^CreERT^ was cross to the R26R^EYFP^ reporter [22], we found a low frequency of labeling in squamous epithelium of both the lingual and buccal surfaces (Figure 1C, D). Because only basal cells are retained for extended lengths of time, we determined the percent of basal cells in lingual and buccal surfaces that were labeled by CK19^CreERT^ and found that 0.82+/−0.42% (n = 3) were positive for the EYFP reporter. Other tissues including the stomach, small intestine, colon, pancreas and liver were labeled as previously described [17].

**Figure 2:**
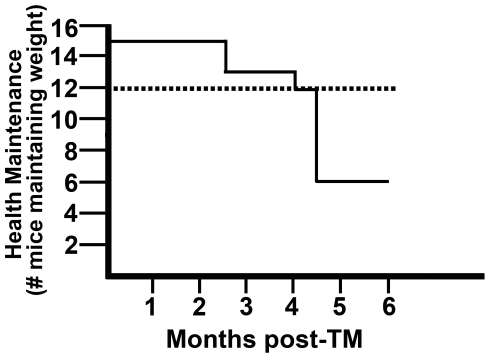
Morbidity analysis: epithelial expression of Kras^G12D^ led to weight loss. Mice were monitored weekly for weight and other indications of overall health. Number maintaining at least 80% of highest body weight is plotted as a function of time for CK19^CreERT^; LSL-Kras^G12D^ (solid line) and for littermates also injected with tamoxifen (dashed line). An additional 3 control mice were also injected with tamoxifen but were only followed for 3–5 months as controls for earlier timepoints and did not lose weight during that time (data not shown).

Fifteen CK19^CreERT^; LSL-Kras^G12D^ mice and littermate controls were given tamoxifen at 6–8 weeks of age and then followed for up to 6 months. Mice were monitored weekly for signs of distress including weight loss. Over the six month time period, 9 of 15 CK19^CreERT^; LSL-Kras^G12D^ mice were euthanized due to loss of at least 20% body weight (Figure 2). No weight loss or other distress was observed in any mouse that had only one of the CK19^CreERT^ or the LSL-Kras^G12D^ alleles.

### Kras^G12D^ mutation induced oral papillomas

**Figure 3:**
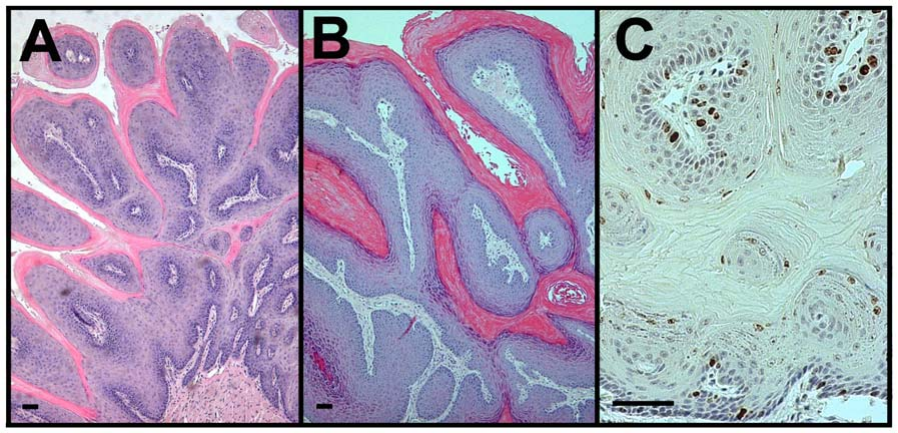
Kras^G12D^ led to squamous papillomas in the oral cavity. A.H&E staining of papillomas on back of tongue. B. H&E staining of buccal papillomas. C. Immunolabeling for phosphohistone H3 (brown), an M phase marker, revealed that the proliferative zone in papillomas remained in the basal layer. Size bars, 50 µm.

Loss of weight was correlated with the presence of large masses in the oral cavity. Histological analyses revealed the presence of squamous papillomas of the posterior tongue and/or buccal surface of all mice that lost >20% body weight (Figure 3A, B). Papillomas of the posterior tongue were found in 100% percent of CK19^CreERT^; LSL-Kras^G12D^ mice while these lesions less commonly involved the buccal surface, occurring in seven of thirteen bigenic mice aged 4–6 months post-tamoxifen treatment. Interestingly, lingual papillomas were limited to the back of the tongue even though CK19^CreERT^ labeled cells randomly across the anterior-posterior axis of the tongue. The proliferative zone in all papillomas was maintained in the basal cell layer (Figure 3C) and no dysplasia was observed. No oral cavity phenotype resulted from tamoxifen injection or from heterozygosity of the CK19 or Kras alleles, as all mice with single genetically engineered alleles injected with tamoxifen had normal phenotype in all tissues examined.

### Kras^G12D^ mutation induced lung adenoma formation

**Figure 4:**
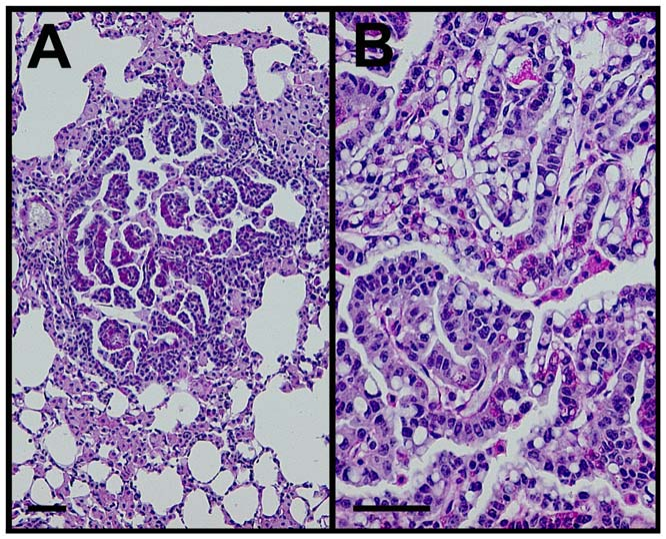
Kras^G12D^ mutation led to adenoma formation in lungs. A, B. PAS and hematoxylin staining of lung adenomas typical of CK19^CreERT^; LSL-Kras^G12D^ mice. Size bars, 50 µm.

At no time during the study was any difficulty in breathing observed in mice even though we found CK19^CreERT^ was active in lung bronchi and bronchioles. However, upon dissection, lungs of CK19^CreERT^; LSL-Kras^G12D^ mice were enlarged with an average wet weight of 0.676+/−0.053g compared to 0.175+/−0.014 g for control lungs at 6 months post-tamoxifen (*p*<0.001; n = 5 CK19^CreERT^; LSL-Kras^G12D^, 9 single transgene controls). All CK19^CreERT^; LSL-Kras^G12D^ mice at 6 months post-tamoxifen had visible tumors up to 3 mm in diameter. Upon histological analysis, all CK19^CreERT^; LSL-Kras^G12D^ mice had multiple benign lung tumors consisting primarily of papillary adenomas that became densely packed in the larger lesions (Figure 4A, B) [23] while all single gene controls were histologically normal.

### Kras^G12D^ mutation induced gastric metaplasia

**Figure 5:**
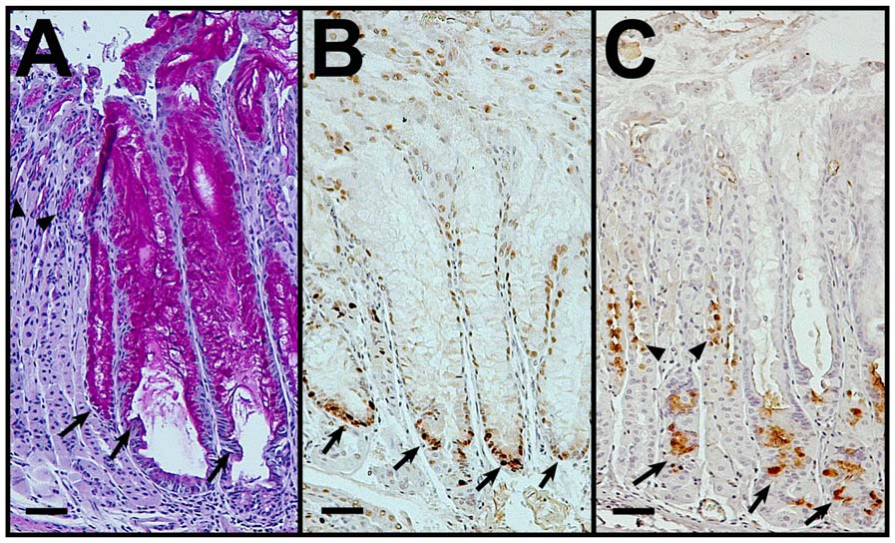
Kras^G12D^ mutation led to foveolar hyperplasia in the gastric fundus. A. PAS (pink) showed extensive mucin production deep into affected glands while surrounding normal glands only had staining at the tops of glands. B. Immunolabeling for phosphohistone H3 as an M phase marker. The proliferative zone in affected glands was shifted toward the base of the glands. C. Immunolabeling for TFF2 (brown) showed positive cells near base of affected fundic glands while normal glands had expression in the normal mucous neck region. Arrows, staining in affected glands; arrowheads, staining in normal glands. Size bars, 50 µm.

All CK19^CreERT^; LSL-Kras^G12D^ mice exhibited metaplasia in the fundus of the stomach by 4–6 months after tamoxifen administration while single gene controls exposed to tamoxifen were morphologically and histologically normal. The number of affected glands in CK19^CreERT^; LSL-Kras^G12D^ mice ranged from 10–50% along the lesser curvature of the stomach with a decreasing frequency farther from the lesser curvature. Affected glands were characterized by foveolar hyperplasia as seen by the extended zone of PAS staining for mucin (Figure 5A), reduced presence of parietal cells (data not shown) and a proliferative zone deeper in the affected glands than in unaffected glands (Figure 5B). TFF2, a marker of mucous neck cells, also showed staining deeper in the glands, indicating that the mucous neck region was located near the base rather than the normal location approx. two-thirds the distance from the base (Figure 5C). Staining with alcian blue yielded a light diffuse staining rather than the dark, punctate pattern seen in intestinal metaplasia (data not shown).

### Kras^G12D^ mutation led to pancreatic ductal lesions

**Figure 6:**
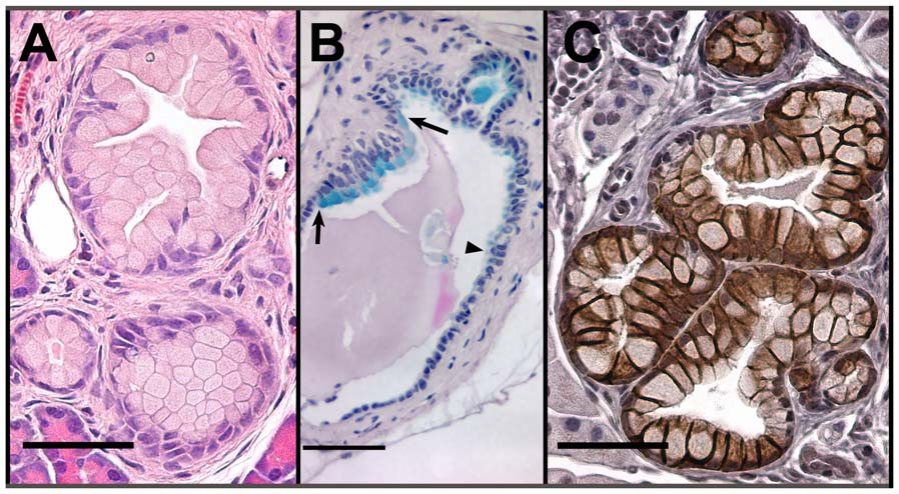
Kras^G12D^ mutation led to mucinous metaplasia in pancreatic ducts. H&E staining of a typical lesion in mouse pancreas (A) and its connection to normal ductal epithelium in a nearby serial section (B) with alcian blue staining to denote mucin-producing cells. Arrowhead, junction between nonmucinous and mucinous cells; arrows, junction between cuboidal and columnar cells. (C) Mucinous lesions were all strongly positive for claudin-18 (brown) with highest concentrations along lateral cell borders. Size bars, 50 µm.

Within the pancreas, CK19^CreERT^ recombines loxP-flanked alleles in ductal cells with less than 1% recombination in islet and acinar cells [17]. Therefore, this allele allowed us to test the hypothesis that ducts could be the origin of pancreatic ductal adenocarcinoma (PDAC) [13]. At dissection, the pancreas appeared grossly normal in CK19^CreERT^; LSL-Kras^G12D^ mice. To detect any morphological changes, we examined sections every 200 µm through each pancreas. All five bigenic mice examined at 6 months of age exhibited at least one mucinous ductal metaplasia with features of early mouse pancreatic intraepithelial neoplasm, or mPanIN1A, thought to be the earliest stage of neoplasm leading to PDAC (Figure 6A) while control littermates were morphologically and histologically normal. Of the 6 bigenic mice examined at 4.5 months post-tamoxifen treatment, two had these mucinous ductal lesions (data not shown). Six of all lesions were found in the tail, three in the body, and two in the head of the pancreas. Examination of serial sections showed that all lesions were continuous with normal pancreatic ducts (Figure 6B) suggesting that they did indeed arise via activation of Kras in pancreatic ducts. At the interface between morphologically normal and abnormal cells, staining with alcian blue demonstrated that morphologically normal cells near the transition site expressed low levels of mucin whereas alcian blue staining was not observed in any normal ducts not associated with ductal lesions. Thus, a progression could be observed from cuboidal, non-mucinous to cuboidal, mucinous to columnar, mucinous cells within one duct. We also determined whether pancreatic lesions in CK19^CreERT^; LSL-Kras^G12D^ mice expressed claudin-18, which is highly expressed in PanINs and PDAC but not in normal ducts or reactive ducts that are commonly seen in chronic pancreatitis [24]. As shown in Figure 6C, all mucinous lesions found expressed claudin-18. No other cells in the pancreas including normal ducts had detectable claudin-18 (data not shown). The ducts that were contiguous with mucinous lesions were all the larger interlobular or intralobular ducts that express the CK19^CreERT^ allele. Because CK19^CreERT^ does not produce Cre recombinase activity in the smaller intercalated ducts, we could not determine whether all ducts of the pancreas could give rise to PanIN-like lesions but can clearly conclude that larger ducts are capable of giving rise to mucinous neoplasms characteristic of early PanIN lesions.

### Kras^G12D^ mutation induced metaplasia in the colon

**Figure 7:**
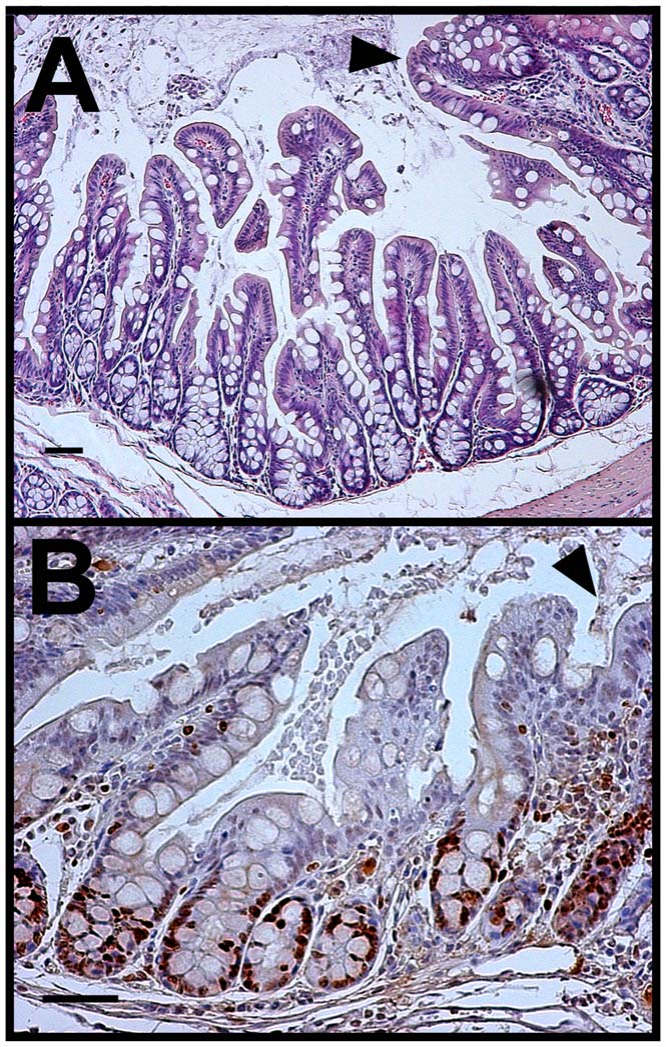
Kras^G12D^ mutation was associated with patches of villus-like structures in the ascending colon. A. H&E staining shows villus-like architecture in an area of the ascending colon. Normal colonic crypt structure resumes on upper right side of each panel (arrowhead). B. Immunolabeling for Ki67 (brown) indicates that the proliferative zones associated with villus-like structures are similar in cell number to normal colonic crypts. Size bars, 50 µm.

In many patches of the ascending colon, villus-like structures were observed surrounded by areas of normal crypt structure in CK19^CreERT^; LSL-Kras^G12D^ mice (Figure 7A). Similar but less distinct villus-like projections were also seen in smaller numbers (1–3 per section) in more distal parts of the colon (data not shown). These villus-like projections did not have apparent Paneth cells and so may not have been a homeotic transformation to small intestine. The tops of projections were approximately the same height as surrounding crypts and they were not associated with increased proliferation (Figure 7B). These structures are morphologically similar to those recently reported as murine serrated hyperplasia capable of giving rise to serrated carcinoma in a mouse model [25]. Single gene control mice had no intestinal phenotype.

### Some tissues appeared unaffected by Kras^G12D^ activation

We have reported previously that the CK19^CreERT^ allele is active in hepatic ducts, renal papillary ducts and throughout the small intestine. However, upon examination of random sections through each of these tissues in each CK19^CreERT^; LSL-Kras^G12D^ mouse at 4–6 months post-tamoxifen, we failed to find any morphological abnormalities in liver, kidney or small intestine. Because CK19^CreERT^ has a high rate of recombination in the small intestine but no morphological phenotype, we also immunolabeled this tissue for markers of proliferation, phospho-histone H3 and Ki67. All intestinal crypts had a normal number of proliferating cells (data not shown). Thus, Kras^G12D^ mutation alone is not sufficient to alter homeostasis in this tissue.

**Figure 8:**
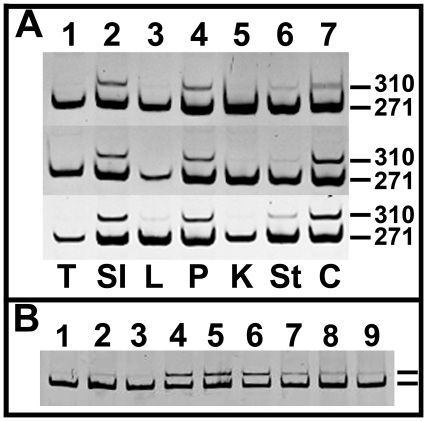
Cre-mediated recombination of the LSL-Kras^G12D^ allele in different tissues. A. DNA was extracted from the indicated tissues of three different mice and subjected to 35 cycles of PCR that would detect both the wildtype Kras allele (271 bp) and the recombined LSL-Kras^G12D^ allele (310 bp) but not the unrecombined LSL-Kras^G12D^ allele. Tissues examined: 1) tail, 2) small intestine, 3) liver, 4) pancreas, 5) kidney, 6) stomach, and 7) colon. Strong recombined bands were detected for small intestine, pancreas and colon and weaker bands for stomach, kidney and liver, while recombination was never detected in tail DNA. B. The amount of recombination of an unaffected tissue, the small intestine, was compared to the amount in an oral papilloma in which all or nearly all of the epithelium should have a recombined Kras allele. For three different mice, DNA was prepared from total oral tissue (lanes 1–3), isolated papilloma tissue (lanes 4–6) and total small intestine tissue (lanes 7–9) and subjected to 30 cycles of PCR. Little recombination can be detected in total oral tissue while the recombination is abundant in the isolated papilloma (upper band in each lane). The amount of recombination in the small intestine was intermediate between these two populations.

One reason for tissue-specific differences in Kras^G12D^ susceptibility could be a difference in the extent of recombination of the LSL-Kras^G12D^ allele. While we had shown that CreERT produced from the CK19 allele could recombine the R26R^EYFP^ allele in all these tissues, different alleles may have different rates of recombination due to loxP flanking sequences or higher level chromatin structure. Therefore, we performed PCR analysis of DNA extracted from each of the tissues that had little or no phenotype in CK19^CreERT^; LSL-Kras^G12D^ mice, as well as from stomach which had a strong phenotype, and from tail DNA as a negative control. As expected, the Kras^G12D^ allele was recombined in stomach but was also recombined to some extent in small intestine, colon, liver, pancreas, and kidney while no recombination was detected in tail DNA (Figure 8A). The amount of recombination was barely detectable in liver and kidney perhaps due to the small number of duct cells in each of those tissues that had shown recombination of the R26R^EYFP^ allele [17]. Because the intestine had a seemingly high amount of recombination of the Kras^G12D^ allele but no apparent phenotype, we compared the amount of recombined allele present in the small intestine to that in oral papillomas, where the majority of epithelium should have recombined the Kras^G12D^ allele (although there was some submucosa present in which CK19^CreERT^ is not active). As expected, the amount of recombination (upper bands in Figure 8B) was much higher in isolated papillomas than in total oral tissue. The amount of recombination in intestine was higher than in total oral tissue but less than that seen in the papillomas. This is consistent with the observed activity of CK19^CreERT^ being high in intestine but not recombining in every cell. In conclusion, CK19^CreERT^ was able to recombine the Kras^G12D^ allele in multiple epithelial tissues even when no resulting phenotype was observed.

## Discussion

### Environmental impact on Kras^G12D^-initiated tumorigenesis

While the CK19^CreERT^ mouse can activate Kras^G12D^ in many epithelial tissues of the mouse, this activation only induced widespread neoplasia in three tissues, the oral mucosa, stomach, and lung. Of the tissues targeted by CK19^CreERT^, these tissues are among the most exposed to the outside environment. This suggests that environment-induced damage or mutation may cooperate with Kras mutation to promote tumor formation. However, anterior tongue and the small intestine did not develop detectable neoplastic changes in response to Kras^G12D^ mutation. Therefore, environmental exposure cannot be the only factor in susceptibility. Rather, cells must also have intrinsic differences in their response to the Kras^G12D^ mutation.

### Some tissue susceptibility may arise from differences in Kras mutation rate

In two cases, Kras^G12D^ initiated neoplastic changes in tissues that rarely have Kras mutations in corresponding human cancers. In both the oral cavity and stomach, frequent and profound hyperplasia resulted from Kras^G12D^ expression, yet cancers of these tissues in humans rarely carry Kras mutations. No information exists on the actual rate of Kras mutation in normal human tissues and we cannot rule out species-specific differences. However, our results combined with other studies discussed below, suggest that some epithelial tumors or hyperplasias do not carry activating mutations in Kras because of selection for other mechanisms that upregulate Kras activity. This may reflect a lower rate of occurrence of Kras mutation in these tissues or a higher rate of other mutations/events that affect Kras activity.

One of these tissues is the oral mucosa. Caulin et al. [26] also showed that Kras^G12D^ can induce squamous papillomas in oral mucosa, using an inducible Cre transgene regulated by the CK14 promoter to activate the LSL-Kras^G12D^ allele. Although activating mutations in Kras are found in only 4–7% of squamous cell carcinoma of the head and neck (HNSCC) in humans [27], [28], Kras protein is often elevated in this cancer suggesting that its activity plays a critical role in tumor initiation or progression. Supporting this role, a low level of expression of microRNA let-7d and a polymorphism in a let-7 microRNA binding site in the Kras 3′UTR are associated with poor survival from HNSCC [29], [30] and let-7 has been shown to regulate Kras expression [31].

In stomach cancer, there have been reports that Kras may also be regulated by microRNAs and/or by gene amplification [32], [33]. We showed that Kras^G12D^ can induce frequent metaplasia in gastric fundus. These metaplastic glands displayed characteristics of foveolar hyperplasia with an extended zone of PAS-positive surface mucous cells, oxyntric atrophy, and basal expression of TFF2. Foveolar hyperplasia is associated with conditions such as Menetrier's disease rather than gastric cancer [34]. It will be interesting in future studies to determine whether these Kras-initiated hyperplasias in stomach mucosa can progress to invasive or metastatic disease.

### Cell of origin in lung and pancreatic cancers

In lungs, the CK19^CreERT^ allele produced recombination mostly in bronchi and bronchioles leading to the papillary adenomas observed in the CK19^CreERT^; LSL-Kras^G12D^ mice. These tumors were quite different than those observed when the LSL-Kras^G12D^ allele was activated by nasal installation of adenovirus encoding Cre recombinase [18], [35]. The difference likely reflects different cells of origin for tumor formation. Since we determined that CK19^CreERT^ is active primarily in bronchi and bronchioles, it is likely that inhaled viral delivery targets mainly other cell types such as alveolar cells.

The cell of origin of pancreatic ductal adenocarcinoma has been widely debated. While these lesions in humans consist entirely of ductal cells, recent studies in mice have pointed to an amazing plasticity in the adult pancreas, showing that acinar cells readily give rise to ductal epithelium, notably to mucinous, hyperplastic epithelium in the case of transgenic overexpression of the growth factor TGFα [14]. Furthermore, studies have shown that using an acinar-specific CreERT allele to activate Kras^G12D^ results in a low frequency of mPanIN1A lesions by 2 months post-tamoxifen treatment and progression to later stage PanINs as mice age [36], [37]. Similarly, expression of Kras^G12V^ in acinar cells also led to mPanIN and PDAC but only in conjunction with chemically induced pancreatitis [38]. Thus, at least in the mouse, acinar cells are capable of giving rise to PanIN-like lesions and PDAC.

Until now, the tools to direct Kras^G12D^ mutation to pancreatic ducts have been lacking and testing the ductal compartment as the cell of origin for PanIN or PDAC was not possible. We now show, using the CK19^CreERT^ allele to activate the Kras^G12D^ allele, that pancreatic ducts can give rise to lesions with the morphological and molecular characteristics of early PanINs. While these cells have the characteristics of early PanIN lesions, we cannot conclude at this point that they are true “PanINs,” because they did not give rise to PDAC in the course of our analysis. Future studies will determine the ability of these lesions to progress to invasive or metastatic disease when combined with loss of tumor suppressor genes or with induction of pancreatitis.

Because CK19^CreERT^ is expressed in a small percentage of acinar cells [17], it is possible that those few cells gave rise to the lesions reported here. This is unlikely for two reasons. One, our model had recombination in <1% of acinar cells while the acinar-specific Elastase-CreERT: LSL-Kras^G12D^ model had 50% of acinar cells recombined and yet had a similar number of PanIN lesions arise as we did in our study [36]. Since CK19^CreERT^ labels <1% of acinar cells [17], we would expect to see a much lower frequency (∼1/50^th^ the number) of lesions than those seen from elastase-driven activation of Kras^G12D^ if acinar cells were the cell of origin in our study. Two, the PanIN-like lesions resulting from CK19^CreERT^ activation were continuous with normal ductal epithelium. Comparing our results and those described above, we conclude that both duct and acinar cells are capable of giving rise to mucinous lesions with PanIN-like characteristics and therefore potentially to PDAC.

### Some tissues in the adult are refractory to Kras^G12D^-initiated tumorigenesis

We did not observe hyperplasia in the intestinal tract although CK19^CreERT^ is widely expressed in this mucosa [17] and a substantial amount of recombination of the Kras^G12D^ allele was seen (Figure 8). Jacks and colleagues [18], [39] reported that activation of the LSL-Kras^G12D^ allele via the FABP-Cre transgene resulted in extensive hyperplasia along the crypt-villus axis throughout the intestine. Rather, we found only induction of altered crypt architecture in the colon and no phenotype in the small intestine. One difference between that study and ours is the timing of Kras^G12D^ activation. FABP-Cre is active embryonically [40] whereas we did not activate CK19^CreERT^ until 6–8 weeks of age. In the pancreas, embryonic activation of the Kras^G12D^ allele resulted in widespread early neoplasms with occasional progression to more advanced lesions [8]. However, activation in adult acinar cells [36], [37] or adult ducts (reported here) gave only rare lesions unless further injury was induced [38]. A similar phenomenon may occur in the intestine, where Kras^G12D^ in the embryonic epithelium induces changes that result in a higher susceptibility to tumorigenesis. Perhaps adult tissues are intrinsically more refractory to the effects of activated Kras or else the embryonic environment (e.g., the abundant mesenchyme that often regulates epithelial development) promotes oncogenic events that do not necessarily become apparent until after birth.

The studies presented here clearly show that different tissues have different susceptibilities to Kras^G12D^-initiated tumorigenesis. This differential susceptibility is most evident in the small intestine which had a high rate of recombination of the LSL-Kras^G12D^ allele with no apparent resulting phenotype. This could be due to multiple mechanisms. It could be that Kras regulates different signaling pathways in different cells and that some pathways are more oncogenic than others. Supporting differences in Kras function, high level of Kras^G12D^ may induce tumorigenesis in some cells but will induce senescence in other cells [41], [42]. Alternatively, susceptibility may depend upon the level of Kras expression. While Kras expression has been found in all tissues examined [43], [44], [45], its relative expression level among different tissues varies [45]. Those experiments were limited in that they did not look at expression in each cell type, but clearly indicated that Kras expression, while ubiquitous, has some tissue-specific regulation. For example, liver has a lower level of Kras expression than many other tissues while gut has among the highest level of Kras expression. Low expression of Kras may explain the inability of Kras^G12D^ to induce liver tumors but does not explain the inability of Kras^G12D^ to induce intestinal tumors. The activity of oncogenes is frequently abrogated by the expression of tumor suppressor genes. Tissues may vary in which tumor suppressors are expressed, how many are expressed, and how readily they are silenced. It is likely that future studies will uncover multiple mechanisms that vary from tissue to tissue, allowing some cells to maintain homeostasis in the face of Kras activation while others become transformed.

## Materials and Methods

### Mice

All experiments were performed with approval from the Vanderbilt Institutional Animal Care and Use Committee, Animal Welfare Assurance number A3227-01, protocol numbers M/06/078 and M/09/394. CK19^CreERT^ [17], LSL-Kras^G12D^ [18] and R26R^EYFP^ [22] mice were previously published and were bred for at least 8 generations into the C57BL6/J background. At 6–8 weeks of age, mice were administered tamoxifen or corn oil vehicle 3 times over the course of 5 days at a dosage of 1 mg in 50 µl each time. Mice were weighed weekly and euthanized if weight decreased by more than 20%. Fifteen bigenic CK19^CreERT^; LSL-Kras^G12D^ mice were analyzed as well as 15 littermate controls that were wildtype or had either the CK19^CreERT^ or the LSL-Kras^G12D^ allele but not both.

### Histology

Tissues were fixed in 4% paraformaldehyde for 4–5 h at 4°C, except tissues for EYFP detection which were fixed overnight, washed in 70% ethanol and embedded in paraffin after dehydration through a series of increasing ethanol concentrations and Histoclear (National Diagnostics, Atlanta, GA). Paraffin sections were cut at a depth of 5 µm. Antibodies used were rabbit anti-GFP (Novus Biological, Littleton, CO), mouse anti-TFF2 (a kind gift of Nick Wright), rabbit anti-phosphohistone H3 (Millipore, Telecuma, CA), rabbit anti-claudin-18 (Zymed/Invitrogen, Carlsbad, CA) and mouse anti-Ki67 (Novocastra, Newcastle upon Tyne, UK). Mucin was detected by staining with periodic acid-Schiff reagent (Sigma, St. Louis, MO) as per manufacturer's instructions or with Alcian blue, pH 2.5 (Sigma).

### Detection of LSL-Kras^G12D^ recombination

Tissue was scraped from slides and digested with proteinase K in DirectPCR lysis buffer (Viagen Biotech Inc, Los Angeles, CA). Lysates were heat-inactivated, extracted with phenol and chloroform, precipitated with isopropanol, resuspended and 100 ng used in polymerase chain reaction (PCR) as described [26] with primer sequences KRasLox-F2: 5′CAGTGCAGTTTTGACACCAGCT3′, and KRasLox-R1: 5′GCATAGTACGCTATACCCTGTGGA3′ and 30–35 cycles of amplification as indicated.

## References

[1] Hahn WC, Weinberg RA (2002). Rules for making human tumor cells.. N Engl J Med.

[2] Ellis CA, Clark G (2000). The importance of being K-Ras.. Cell Signal.

[3] Luttges J, Reinecke-Luthge A, Mollmann B, Menke MA, Clemens A (1999). Duct changes and K-ras mutations in the disease-free pancreas: analysis of type, age relation and spatial distribution.. Virchows Arch.

[4] Friday BB, Adjei AA (2005). K-ras as a target for cancer therapy.. Biochim Biophys Acta.

[5] Feldmann G, Beaty R, Hruban RH, Maitra A (2007). Molecular genetics of pancreatic intraepithelial neoplasia.. J Hepatobiliary Pancreat Surg.

[6] Vogelstein B, Fearon ER, Hamilton SR, Kern SE, Preisinger AC (1988). Genetic alterations during colorectal-tumor development.. N Engl J Med.

[7] Hansel DE, Kern SE, Hruban RH (2003). Molecular pathogenesis of pancreatic cancer.. Annu Rev Genomics Hum Genet.

[8] Hingorani SR, Petricoin EF, Maitra A, Rajapakse V, King C (2003). Preinvasive and invasive ductal pancreatic cancer and its early detection in the mouse.. Cancer Cell.

[9] Hruban RH, Adsay NV, Albores-Saavedra J, Compton C, Garrett ES (2001). Pancreatic intraepithelial neoplasia: a new nomenclature and classification system for pancreatic duct lesions.. Am J Surg Pathol.

[10] Aguirre AJ, Bardeesy N, Sinha M, Lopez L, Tuveson DA (2003). Activated Kras and Ink4a/Arf deficiency cooperate to produce metastatic pancreatic ductal adenocarcinoma.. Genes Dev.

[11] Hingorani SR, Wang L, Multani AS, Combs C, Deramaudt TB (2005). Trp53R172H and KrasG12D cooperate to promote chromosomal instability and widely metastatic pancreatic ductal adenocarcinoma in mice.. Cancer Cell.

[12] Ijichi H, Chytil A, Gorska AE, Aakre ME, Fujitani Y (2006). Aggressive pancreatic ductal adenocarcinoma in mice caused by pancreas-specific blockade of transforming growth factor-beta signaling in cooperation with active Kras expression.. Genes Dev.

[13] Hruban RH, Goggins M, Parsons J, Kern SE (2000). Progression model for pancreatic cancer.. Clin Cancer Res.

[14] Blaine SA, Ray KC, Anunobi R, Gannon MA, Washington MK (2010). Adult pancreatic acinar cells give rise to ducts but not endocrine cells in response to growth factor signaling..

[15] Means AL, Meszoely IM, Suzuki K, Miyamoto Y, Rustgi AK (2005). Pancreatic epithelial plasticity mediated by acinar cell transdifferentiation and generation of nestin-positive intermediates.. Development.

[16] Strobel O, Dor Y, Alsina J, Stirman A, Lauwers G (2007). In vivo lineage tracing defines the role of acinar-to-ductal transdifferentiation in inflammatory ductal metaplasia.. Gastroenterology.

[17] Means A, Xu Y, Zhao A, Ray K, Gu G (2008). A CK19-CreERT knockin mouse line allows for conditional DNA recombination in epithelial cells in multiple endodermal organs.. Genesis.

[18] Tuveson DA, Shaw AT, Willis NA, Silver DP, Jackson EL (2004). Endogenous oncogenic K-ras(G12D) stimulates proliferation and widespread neoplastic and developmental defects.. Cancer Cell.

[19] Nakajima M, Kawanami O, Jin E, Ghazizadeh M, Honda M (1998). Immunohistochemical and ultrastructural studies of basal cells, Clara cells and bronchiolar cuboidal cells in normal human airways.. Pathol Int.

[20] Schlage WK, Bulles H, Friedrichs D, Kuhn M, Teredesai A (1998). Cytokeratin expression patterns in the rat respiratory tract as markers of epithelial differentiation in inhalation toxicology. I. Determination of normal cytokeratin expression patterns in nose, larynx, trachea, and lung.. Toxicol Pathol.

[21] Moll R, Franke WW, Schiller DL, Geiger B, Krepler R (1982). The catalog of human cytokeratins: patterns of expression in normal epithelia, tumors and cultured cells.. Cell.

[22] Srinivas S, Watanabe T, Lin CS, William CM, Tanabe Y (2001). Cre reporter strains produced by targeted insertion of EYFP and ECFP into the ROSA26 locus.. BMC Dev Biol.

[23] Nikitin AY, Alcaraz A, Anver MR, Bronson RT, Cardiff RD (2004). Classification of proliferative pulmonary lesions of the mouse: recommendations of the mouse models of human cancers consortium.. Cancer Res.

[24] Karanjawala ZE, Illei PB, Ashfaq R, Infante JR, Murphy K (2008). New markers of pancreatic cancer identified through differential gene expression analyses: claudin 18 and annexin A8.. Am J Surg Pathol.

[25] Bennecke M, Kriegl L, Bajbouj M, Retzlaff K, Robine S (2010). Ink4a/Arf and oncogene-induced senescence prevent tumor progression during alternative colorectal tumorigenesis.. Cancer Cell.

[26] Caulin C, Nguyen T, Longley MA, Zhou Z, Wang XJ (2004). Inducible activation of oncogenic K-ras results in tumor formation in the oral cavity.. Cancer Res.

[27] Lea IA, Jackson MA, Li X, Bailey S, Peddada SD (2007). Genetic pathways and mutation profiles of human cancers: site- and exposure-specific patterns.. Carcinogenesis.

[28] Sheu JJ, Hua CH, Wan L, Lin YJ, Lai MT (2009). Functional genomic analysis identified epidermal growth factor receptor activation as the most common genetic event in oral squamous cell carcinoma.. Cancer Res.

[29] Childs G, Fazzari M, Kung G, Kawachi N, Brandwein-Gensler M (2009). Low-level expression of microRNAs let-7d and miR-205 are prognostic markers of head and neck squamous cell carcinoma.. Am J Pathol.

[30] Christensen BC, Moyer BJ, Avissar M, Ouellet LG, Plaza SL (2009). A let-7 microRNA-binding site polymorphism in the KRAS 3′ UTR is associated with reduced survival in oral cancers.. Carcinogenesis.

[31] Johnson SM, Grosshans H, Shingara J, Byrom M, Jarvis R (2005). RAS is regulated by the let-7 microRNA family.. Cell.

[32] Hashimoto Y, Akiyama Y, Otsubo T, Shimada S, Yuasa Y (2010). Involvement of epigenetically silenced microRNA-181c in gastric carcinogenesis.. Carcinogenesis.

[33] Mita H, Toyota M, Aoki F, Akashi H, Maruyama R (2009). A novel method, digital genome scanning detects KRAS gene amplification in gastric cancers: involvement of overexpressed wild-type KRAS in downstream signaling and cancer cell growth.. BMC Cancer.

[34] Coffey RJ, Washington MK, Corless CL, Heinrich MC (2007). Menetrier disease and gastrointestinal stromal tumors: hyperproliferative disorders of the stomach.. J Clin Invest.

[35] Jackson EL, Olive KP, Tuveson DA, Bronson R, Crowley D (2005). The differential effects of mutant p53 alleles on advanced murine lung cancer.. Cancer Res.

[36] De La O JP, Emerson LL, Goodman JL, Froebe SC, Illum BE (2008). Notch and Kras reprogram pancreatic acinar cells to ductal intraepithelial neoplasia.. Proc Natl Acad Sci U S A.

[37] Habbe N, Shi G, Meguid RA, Fendrich V, Esni F (2008). Spontaneous induction of murine pancreatic intraepithelial neoplasia (mPanIN) by acinar cell targeting of oncogenic Kras in adult mice.. Proc Natl Acad Sci U S A.

[38] Guerra C, Schuhmacher AJ, Canamero M, Grippo PJ, Verdaguer L (2007). Chronic pancreatitis is essential for induction of pancreatic ductal adenocarcinoma by K-Ras oncogenes in adult mice.. Cancer Cell.

[39] Haigis KM, Kendall KR, Wang Y, Cheung A, Haigis MC (2008). Differential effects of oncogenic K-Ras and N-Ras on proliferation, differentiation and tumor progression in the colon.. Nat Genet.

[40] Saam JR, Gordon JI (1999). Inducible gene knockouts in the small intestinal and colonic epithelium.. J Biol Chem.

[41] DeNicola GM, Tuveson DA (2009). RAS in cellular transformation and senescence.. Eur J Cancer.

[42] Ji B, Tsou L, Wang H, Gaiser S, Chang DZ (2009). Ras activity levels control the development of pancreatic diseases.. Gastroenterology.

[43] Chesa PG, Rettig WJ, Melamed MR, Old LJ, Niman HL (1987). Expression of p21ras in normal and malignant human tissues: lack of association with proliferation and malignancy.. Proc Natl Acad Sci U S A.

[44] Furth ME, Aldrich TH, Cordon-Cardo C (1987). Expression of ras proto-oncogene proteins in normal human tissues.. Oncogene.

[45] Leon J, Guerrero I, Pellicer A (1987). Differential expression of the ras gene family in mice.. Mol Cell Biol.

